# Herpesvirus Antibodies, Vitamin D and Short-Chain Fatty Acids: Their Correlation with Cell Subsets in Multiple Sclerosis Patients and Healthy Controls

**DOI:** 10.3390/cells10010119

**Published:** 2021-01-10

**Authors:** Maria Inmaculada Dominguez-Mozo, Silvia Perez-Perez, Noelia Villarrubia, Lucienne Costa-Frossard, Jose Ignacio Fernandez-Velasco, Isabel Ortega-Madueño, Maria Angel Garcia-Martinez, Estefania Garcia-Calvo, Hector Estevez, Jose Luis Luque Garcia, Maria Josefa Torrejon, Rafael Arroyo, Luisa Maria Villar, Roberto Alvarez-Lafuente

**Affiliations:** 1 Grupo de Investigación de Factores Ambientales en Enfermedades Degenerativas, Instituto de Investigación Sanitaria del Hospital Clínico San Carlos (IdISSC)/Red Española de Esclerosis Múltiple (REEM), 28040 Madrid, Spain; mariadomomd@gmail.com (M.I.D.-M.); silvip93@hotmail.com (S.P.-P.); isormad@hotmail.com (I.O.-M.); garcia.angel23@gmail.com (M.A.G.-M.); 2 Servicio de Inmunología, Hospital Universitario Ramón y Cajal/Instituto Ramón y Cajal de Investigación Sanitaria (IRYCIS), Red Española de Esclerosis Múltiple (REEM), 28040 Madrid, Spain; noelia.villarrubia@salud.madrid.org (N.V.); jfvelasco@salud.madrid.org (J.I.F.-V.); luisamaria.villar@salud.madrid.org (L.M.V.); 3 Servicio de Neurología, Hospital Universitario Ramón y Cajal/Instituto Ramón y Cajal de Investigación Sanitaria (IRYCIS), Red Española de Esclerosis Múltiple (REEM), 28040 Madrid, Spain; lufrossard@yahoo.es; 4 Department of Analytical Chemistry, Faculty of Chemical Sciences, Universidad Complutense de Madrid, 28040 Madrid, Spain; egcalvo@ucm.es (E.G.-C.); hestevez@ucm.es (H.E.); jlluque@ucm.es (J.L.L.G.); 5 Servicio de Análisis Clínicos, Hospital Clínico San Carlos, 28040 Madrid, Spain; mariajosefa.torrejon@salud.madrid.org; 6 Departamento de Neurología, Hospital Universitario Quironsalud Madrid, Red Española de Esclerosis Múltiple (REEM), 28040 Madrid, Spain; rafaelarroyo09@gmail.com

**Keywords:** human herpesvirus, vitamin D, multiple sclerosis, ELISA, flow-cytometry

## Abstract

Although the etiology of multiple sclerosis (MS) is still unknown, it is commonly accepted that environmental factors could contribute to the disease. The objective of this study was to analyze the humoral response to Epstein-Barr virus, human herpesvirus 6A/B and cytomegalovirus, and the levels of 25-hydroxyvitamin D (25(OH)D) and the three main short-chain fatty acids (SCFA), propionate (PA), butyrate (BA) and acetate (AA), in MS patients and healthy controls (HC) to understand how they could contribute to the pathogenesis of the disease. With this purpose, we analyzed the correlations among them and with different clinical variables and a wide panel of cell subsets. We found statistically significant differences for most of the environmental factors analyzed when we compared MS patients and HC, supporting their possible involvement in the disease. The strongest correlations with the clinical variables and the cell subsets analyzed were found for 25(OH)D and SCFAs levels. A correlation was also found between 25(OH)D and PA/AA ratio, and the interaction between these factors negatively correlated with interleukin 17 (IL-17)-producing CD4+ and CD8+ T cells in untreated MS patients. Therapies that simultaneously increase vitamin D levels and modify the proportion of SCFA could be evaluated in the future.

## 1. Introduction

Multiple sclerosis (MS) is an inflammatory demyelinating disease of the central nervous system (CNS). Although the cause of MS is still unknown, it is thought to be an autoimmune disease in which the body’s immune system attacks its own tissues. It is also commonly accepted that an infectious agent could trigger the autoimmune reaction in genetically predisposed subjects [1]. Viruses have been involved in this pathology by an extent number of published studies, mainly those of the *Herpesviridae* family [2,3]. But, also, other environmental factors, such as sun exposure, hypovitaminosis D, smoking or more recently the microbiota, have been related to the pathogenesis of the disease as possible modulators of the immune system in MS patients [4,5,6]. All these environmental factors would have the ability of changing the proportion of different cell subsets, leading to aberrant immune processes in the disease.

There are different publications that have analyzed possible associations between viruses and immune cells in recent years. Thus, regarding Epstein-Barr virus (EBV), that has been repeatedly involved in the pathogenesis of MS, it has been published that IFN-gamma-producing T-cells increased their frequency in patients with clinically isolated syndrome (CIS) after stimulation with Epstein-Barr nuclear antigen 1 (EBNA1) [7]. Another virus related to MS is cytomegalovirus (CMV). Despite the controversial results, the serological status against this virus was found associated with the risk of MS through the induction of differentiated T- and NK-cell subsets [8]. In another paper of the same group, authors showed that CMV seropositivity was associated with greater proportions of NKG2C(+) NK cells, with no significant differences between MS patients and controls [9].

Associations between immune cells and other environmental factors have also been reported. Although hypovitaminosis D has been repeatedly observed in MS patients, association studies with cells of the immune system have had ambiguous results. An open-label, randomized, prospective controlled 52-week trial in patients with MS treated with escalating vitamin D doses up to 40,000 IU/day over 28 weeks showed that T-cell reactivity and proliferation dropped significantly in treated patients over the treatment period, while no change was seen in controls [10]. However, a sub-study of a larger clinical trial exploring high-dose (up to 14,000 IU/day) vitamin D supplementation, as an add-on therapy to interferon beta 1a in patients with relapsing-remitting MS (RRMS), showed no difference in either IL-17 CD4+ or IFN-gamma CD4+ T cells at 48 weeks of observation [11].

In recent years, the intestinal flora has been reported to be closely linked to various autoimmune diseases. With regard to MS, patients with this disease seem to exhibit gut microbial dysbiosis [12]. Since metabolic products of bacteria, such as short-chain fatty acids (SCFAs), seem to play an important role in immune disorders, including MS, their evaluation could aid to understand the aberrant immune processes in this disease. Thus, fecal SCFAs, acetate (AA), propionate (PA) and butyrate (BA), were found depleted in MS patients in comparison with healthy controls (HC); furthermore, the concentration of fecal SCFAs was positively correlated with the proportion of regulatory T (Treg) cells [13]. But these metabolites can also be measured in serum/plasma. They can reach the blood stream passing through the intestinal barrier and then interact with immune cells or with those cells carrying receptors for them. Thus, it has been described that the ratio between butyrate and caproic acid (another SCFA) correlated positively with CD4+/CD25high/Foxp3+ and negatively with CD4+/IFNγ+ T lymphocytes [14]. In a supplementation study of therapy-naive MS patients, PA was associated with a significant and sustained increase of functionally competent Treg cells, whereas Th1 and Th17 cells decreased significantly [15].

However, in most cases, these environmental factors have been studied separately and there are no results about their possible interactions. With this purpose, we analyzed the humoral response to EBV, human herpesvirus 6A/B (HHV-6A/B) and CMV, and the levels of 25-hydroxyvitamin D (25(OH)D) in serum (despite not being active, 25(OH)D is used to evaluate vitamin D serological levels) [16] and the three main SCFA (PA, BA and AA) in MS patients and healthy controls (HC), and the correlations among them with different clinical variables and with a wide panel of cell subsets were assessed. The results of the study showed statistically significant differences for most of the environmental factors analyzed when we compared MS patients and HC, supporting their possible involvement in the disease. The strongest correlations with the clinical variables and the cell subsets analyzed were found for 25(OH)D and SCFAs levels. A correlation was also found between 25(OH)D and PA/AA ratio—the interaction between these factors negatively correlated with IL-17-producing CD4+ and CD8+ T cells in untreated MS patients.

## 2. Materials and Methods

### 2.1. Subjects

A total of 191 MS patients, 133 with relapsing-remitting MS (RRMS) and 58 with secondary progressive MS (SPMS), diagnosed by Poser [17] or McDonald’s [18] 2010 criteria, and 79 HC recruited from “Hospital Universitario Ramón y Cajal” and “Hospital Clínico San Carlos” were included in the study. All clinical data were collected by neurologists of the Multiple Sclerosis Units of those hospitals (Table 1).

### 2.2. Collection of Samples

All samples were collected between 8 and 11 AM. Two cell preparation tubes with sodium citrate (CPTTM, BD Vacutainer^®^) were collected for cell and plasma isolation. Peripheral blood mononuclear cells (PBMCs) were isolated using density gradient centrifugation (920 g, 30 min): they were cryopreserved in fetal bovine serum (FBS) with DMSO (10%) and stored in liquid nitrogen (−196 °C). Serum was obtained by vein puncture and isolated by centrifugation (920 g, 15 min, room temperature) in serum separator tubes. After centrifugation, plasma and serum samples were aliquoted and stored at −80 °C.

### 2.3. Liquid Chromatography-Mass Spectrometry (LC-MS/MS) Analysis

A 8030 Shimadzu triple-quadrupole mass spectrometer equipped with an ESI ionization source and operating in negative mode was used for the determination of the levels of AA, PA and BA in plasma samples. The chromatographic separation was performed using a Phenomenex Gemini C18 (5 µm, 150 × 2 mm) column, using 0.01% formic acid in water (solvent A) and 0.01% formic acid in acetonitrile (solvent B). The gradient program was as follows: 20% phase B was used as initial mobile phase for 2 min. Two linear gradients were established in order to reach first, a 40% phase B composition in 5 min and then, a final 100% phase B composition in 0.5 min. 100% phase B was maintained for 0.5 min and finally, 2 min were necessary for re-establishing the initial conditions. Samples were delivered at a flow rate of 0.6 mL min^−1^. Column temperature was set to 40 °C and autosampler temperature was set to 4 °C. A sample volume of 10 µL was analyzed in all cases.

The multiple reaction monitoring (MRM) transitions were selected by direct infusion into the mass spectrometer of a mixture of the 3 derivative compounds at 5 mg L^−1^. Mass range for full-mass Q1 scans was set between 100 and 1000 m/z using a 0.309 s scan time. Optimization of the collision energy for the precursor ions was carried out by ramping from 10 to 120 V in the collision cell. Fragment ions were detected in Q3 obtaining Q1/Q3 pairs for each analyte. Nitrogen gas was employed as nebulizing (1.5 L min^−1^) and drying gas (15 L min^−1^), while argon served as collision gas (17 kPa). ESI capillary voltage was set to 4.5 kV and the DL temperature to 250 °C. Data were obtained and processed with LabSolution software. One of the MRM transitions was used for quantitation and the other one acted as a qualifier for verifying the compound identity.

### 2.4. ELISA Analysis

Every serum sample was tested with commercial tests for the detection of anti-HHV-6A/B IgG and IgM (Vidia, Ltd., Czech Republic), anti-EBNA-1 and anti-VCA IgG (Trinity Biotech, USA), and IgG and IgM against CMV (Vircell, Spain), following manufacturers’ instructions, in an automated ELISA processing system (DS2, Dynex Technologies, Chantilly, VA, USA). Results were expressed in artificial units (AU): they were calculated by multiplying the index value by 10 (index value = sample absorbance/cut-off value). Samples were analyzed in duplicate for each test, and doubtful samples, those that were between 9 and 11 AU, were tested again. If the re-tested samples were below 11 AU, they were considered as negative.

### 2.5. 25-Hydroxyvitamin D (25(OH)D) Determination

25(OH)D levels were analyzed by chemiluminescent microparticle immunoassay (CMIA) (Abbot, Wiesbaden, Germany), following the manufacturer’s instructions. All samples were analyzed in the Department of Clinical Analysis of Hospital Clínico San Carlos, at the same time, and using the same device.

### 2.6. Monoclonal Antibodies

For the study of the cell populations, the following monoclonal antibodies were used: Granulocyte/macrophage-colony stimulating factor (GM-CSF)-PE, CD197-PE (CCR7-PE), CD24-PE, Interferon (IFN)-gamma-FITC, CD14-FITC, CD16-PE-Cy7, CD8-FITC, CD27-FITC, CD8-APC-H7, CD4-APC-H7, CD4-PE-Cy7, CD123-Bv421, CD56-APC, CD56-Bv421, CD45RO-APC, CD45-APC-H7, CD3-BV421, CD127-BV421, CD127-FITC, CD45-V500 TNF-alpha-PerCP-Cy5.5, CD38-PE-Cy5.5, CD19-PE-Cy7, CD19-APC, CD25-PE-Cy7, CD3-PerCP, HLA-DR-PerCP, CD11c-PE (BD Biosciences, San Diego, CA, USA) and IL-17-APC (R&D Systems, Minneapolis, MN, USA).

### 2.7. In Vitro Stimulation and Labelling of Antigens

Briefly, 10^6^ PMBCs were labelled with monoclonal antibodies for membrane antigen staining; then, PBMCs were washed with PBS and finally analyzed in a flow cytometer (FACSCanto II. BD Biosciences). Intracellular cytokine detection was performed stimulating 10^6^ PMBCs with 50 ng/mL Phorbol 12-myristate 13-acetate (PMA) and 750 ng/mL Ionomycin (Sigma-Aldrich, St. Louis, MO, USA), in the presence of 2 µg/mL Brefeldin A and 2.1 µM Monensin (BD Biosciences), during 4 h. After the cells were stained with monoclonal antibodies recognizing the surface antigens, PBMCs were fixed and permeabilized with a Cytofix/Cytoperm Kit (BD Biosciences) and stained intracellularly with monoclonal antibodies recognizing IL-17, IFN-gamma, TNF-alpha and GM-CSF cytokines. Cells were finally analyzed in a flow cytometer (FACSCanto II. BD Biosciences).

### 2.8. Flow Cytometry

**Figure 1:**
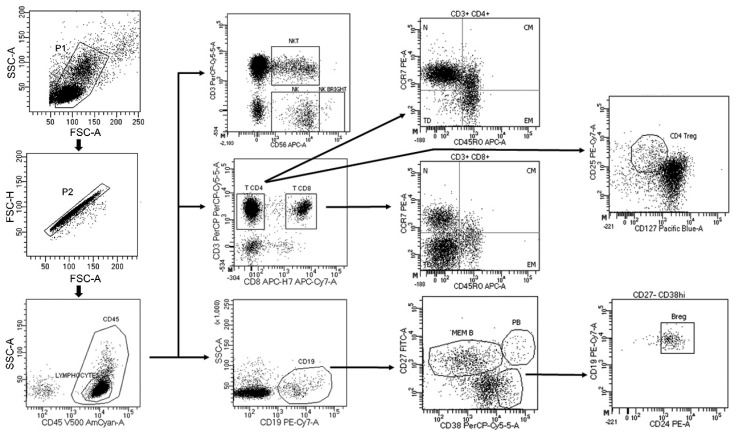
Gating strategy.

A gate including lymphocytes and monocytes and excluding debris and apoptotic cells was first established (Figure 1). CD4+ and CD8+ T cells were classified as: central memory (CM) (CCR7+ CD45RO+), naïve (CCR7+ CD45RO-), terminally differentiated (TD) (CCR7- CD45RO-) and effector memory (EM) (CCR7- CD45RO+). Regulatory CD4 T cells (Treg) were defined as CD3+ CD4+ CD25hi CD127low. B cells were classified as: plasmablasts (CD19+ CD27hi CD38hi), memory (CD19+ CD27dim CD38dim) and regulatory B cells (Breg) (CD19+ CD27- CD24hi CD38hi). Monocytes were also studied, and their subsets were classified according to the expression of CD14 and CD16 (Mo1: CD14++ CD16-; Mo2: CD14++ CD16+; Mo3: CD14+ CD16+). Dendritic cells (DCs) were differentiated into myeloid DCs (mDC: CD14- CD11c+) and plasmacytoid DCs (pDC: HLA-DR+ CD123+). Natural killer (NK) cells (CD56dim CD3-), natural killer T (NKT) cells (CD56dim CD3+) and CD56bright NK cells (CD3- CD56bright) were also studied. Data analysis was performed using FACSDiva Software V.8.0 (BD Biosciences).

### 2.9. Clinical Data

The following clinical variables were considered for analysis at sample collection: starting age (years), MS disease duration (months), Expanded Disability Status Scale (EDSS) score and Multiple Sclerosis Severity Score (MSSS), annualized rate of relapses from the beginning of the disease, number of relapses two years earlier and current treatment.

### 2.10. Statistical Analysis

Continuous variables were expressed as median (25th, 75th percentile) and categorical variables as percentages. Categorical variables were compared using the chi-squared test or Fisher’s exact test, and continuous variables between groups were compared using the Mann–Whitney U-test and the Spearman’s rank correlation coefficient was used when studying the relationship between two continuous variables, including the percentages of the different immune populations. When necessary, the Bonferroni adjustment was performed. Subjects with missing data were omitted from the corresponding analyses. *p*-values ≤ 0.05 were referred to as statistically significant in the text. Analyses were performed using SPSS for Windows (Ver. 15.0) software (SPSS Inc, Chicago, IL, USA) and GraphPad Prism 6.0 software (GraphPad Prism Inc, La Jolla, CA, USA).

## 3. Results

### 3.1. Environmental Factors Included in the Study: Healthy Controls vs. MS Patients

When we analyzed the prevalence of the IgG and IgM antibodies against the viruses included in the study, we found the results shown in Table 2. As can be seen, we only found statistically significant differences for EBNA-1 IgG (higher in MS patients) and CMV IgG (higher in HC).

**Figure 2:**
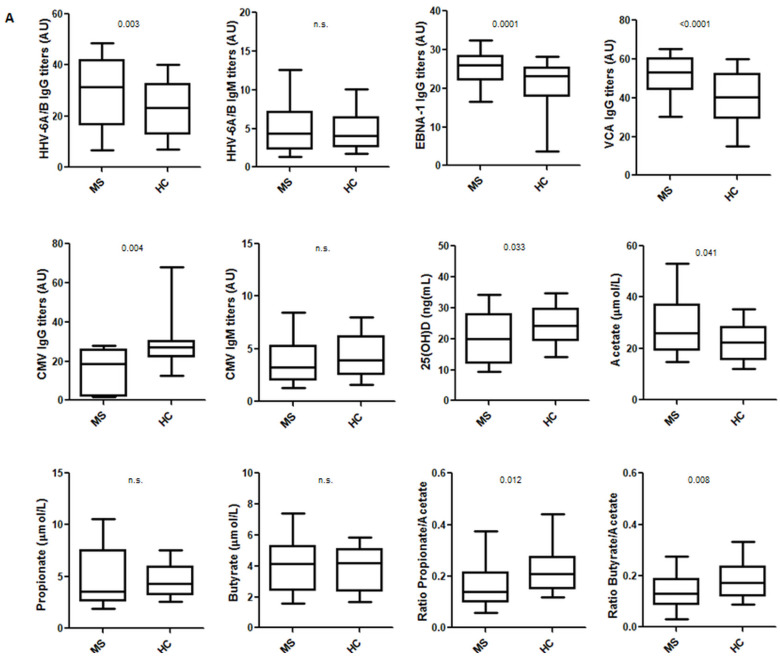

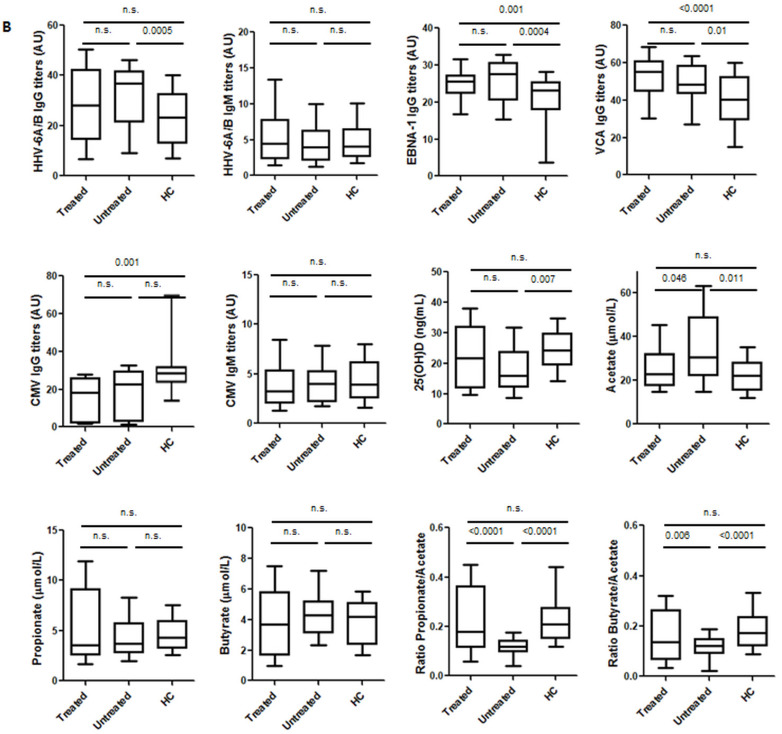
(**A**) Comparisons of the titers of the IgG and IgM viral antibodies, the levels of 25(OH)D, the levels of the three SCFA and PA/AA and BA/AA ratios between MS patients and HC. (**B**) MS patients were divided into those who were receiving treatment and those who were not at the time of sample collection. The upper line shows the comparison between treated MS patients and HC, the lower left line shows the comparison between treated and untreated MS patients and the lower right line shows the comparison between untreated MS patients and HC. Significant differences with the two-tailed *t*-test are shown (n.s.: not significant). Line inside the box shows the median value.

However, when we analyzed the titers of the IgG and IgM antibodies against those viruses, we found statistically significant differences for HHV-6A/B IgG, EBNA-1 IgG, VCA IgG (all of them higher in MS patients) and CMV IgG (higher in HC) (Figure 2A). The comparison of the levels of the other environmental factors included in the study between MS patients and HC showed statistically significant differences for 25(OH)D (higher in HC) and AA (higher in MS patients). Regarding SCFA, while PA and BA have a known anti-inflammatory effect [15,19], it has been suggested that AA could activate the immune cells [20]. Thus, we decided to analyze also the ratio PA/AA and BA/AA; as we can see in Figure 2A, both ratios were significantly higher in HC. Although a statistically significant difference was found for 25(OH)D, due to its seasonal distribution, we also compared the levels of MS patients and HC in the first and second semester of the year: we only found a significant difference for the second semester of the year (23.3 ng/mL vs. 26.3 ng/mL, respectively; *p* = 0.043). The percentage of MS patients with levels of 25(OH)D < 10 ng/mL was 15.7% (16/102) vs. 1.8% (1/57) among HC (*p* = 0.006).

As it has been published that treatments in MS can modify the levels of the different environmental factors, we also analyzed this effect in our MS patients. Regarding the prevalence of the IgG and IgM antibodies against the viruses analyzed, we did not find any statistically significant difference when we compared treated and untreated MS patients: 86.6% vs. 90.9% for HHV-6A/B IgG, 16.7% vs. 7.5% for HHV-6A/B IgM, 94.6% vs. 94.6% for EBNA-1 IgG, 99.2% vs. 100% for VCA IgG, 61.4% vs. 61.4% for CMV IgG and 6.4% vs. 0% for CMV IgM, respectively. Comparisons of the titers of the IgG and IgM viral antibodies and for the levels of the other environmental factors between treated, untreated and HC are shown in Figure 2B. Untreated MS patients showed significantly higher levels of HHV-6A/B IgG (*p* = 0.0005), EBNA-1 IgG (*p* = 0.0004), VCA IgG (*p* = 0.010) and AA (*p* = 0.011) in comparison with HC, but significantly lower levels of 25(OH)D (*p* = 0.007) and PA/AA and BA/AA ratios (*p* < 0.0001 in both cases).

Finally, we also analyzed these environmental factors in RRMS and SPMS patients, and the comparisons with HC are shown in Supplementary Figure S1. Statistically significant differences were found for EBNA-1 and VCA IgG titers and both ratios (PA/AA and BA/AA) when we compared RRMS and SPMS patients with HC. However, HHV-6A/B IgG titers were higher only in RRMS patients in comparison with HC (*p* = 0.0008), CMV IgG titers were lower only in RRMS patients (*p* = 0.0008), 25(OH)D levels were significantly lower only in SPMS patients (*p* = 0.0004) and acetate levels were higher only in SPMS patients (*p* = 0.0006).

### 3.2. Correlations among the Environmental Factors Included in the Study

#### 3.2.1. Correlations among the Environmental Factors in Healthy Controls

As we can see in Table 3, we analyzed the possible relationship between pairs of environmental factors previously related with MS pathogenesis. Among HC, we found a strong correlation between HHV-6A/B IgM and CMV IgM (*r* = 0.623; *p* < 0.0001). The three SCFA also correlated among them, but they did not correlate with any of the other variables (a trend was found for 25(OH)D and BA/AA ratio (*r* = 0.406, *p* = 0.009)).

#### 3.2.2. Correlations among the Environmental Factors in MS Patients

Among MS patients, we found a correlation between HHV-6A/B IgG and IgM (*r* = 0.272; *p* = 0.0002) that was not previously found in the HC group (Table 4). The three SCFA also correlated among them again, and the level of significance was higher than in the HC group. Furthermore, a correlation between the levels of 25(OH)D and the PA/AA ratio not found in the HC group was also obtained (*r* = 0.365, *p* = 0.0003).

### 3.3. Correlations among the Environmental Factors Included in the Study with the Demographic and Clinical Data

#### 3.3.1. Correlations among Environmental Factors with Gender and Age in Healthy Controls

We did not find any association among the environmental factors analyzed with the gender or the age of the HC included in the study (Supplementary Table S1).

#### 3.3.2. Correlations among Environmental Factors with Demographic and Clinical Data in Treated and Untreated MS Patients

Regarding gender and age, we found a statistically significant difference for the HHV-6A/B IgM titers between males and females (3.2 vs. 4.9 AU, respectively; *p* = 0.033), and a trend was also found for HHV-6A/B IgG titers (26.1 vs. 34.1 AU, respectively; *p* = 0.065). We also found a correlation between CMV IgG titers and the age of the MS patients (*r* = 0.255; *p* = 0.002) (Supplementary Table S2).

When we analyzed the possible correlations among the environmental factors and the clinical variables included in the study, we found the following statistical correlations after Bonferroni corrections: 25(OH)D with EDSS (*r* = −0.372; *p* = 0.0001) and MSSS (*r* = −0.402; *p* < 0.0001), AA with EDSS (*r* = 0.363; *p* = 0.0003) and disease duration (*r* = 0.309; *p* = 0.002) and HHV-6A/B IgG titers with MSSS (*r* = −0.198; *p* = 0.007) (Supplementary Table S3). When we analyzed separately treated and untreated MS patients (see Supplementary Tables S4 and S5), we found the following correlations among the untreated MS patients: 25(OH)D with EDSS (*r* = −0.417; *p* = 0.003) and MSSS (*r* = −0.465; *p* = 0.0009), AA with EDSS (*r* = 0.393; *p* = 0.005), PA with disease duration (*r* = 0.446; *p* = 0.002), and AA (*r* = −0.386; *p* = 0.006), PA (*r* = −0.429; *p* = 0.003) and BA (*r* = −0.395; *p* = 0.007) with the starting age. Among treated MS patients, after Bonferroni corrections, we only found the following significant correlations: HHV-6A/B IgG titers with MSSS (*r* = −0.264; *p* = 0.003) and between VCA IgG titers and the starting age (*r* = −0.238; *p* = 0.006).

### 3.4. Correlations among the Environmental Factors Included in the Study and the Immune Cells

#### 3.4.1. Correlations with Immune Cells in Healthy Controls

**Figure 3:**
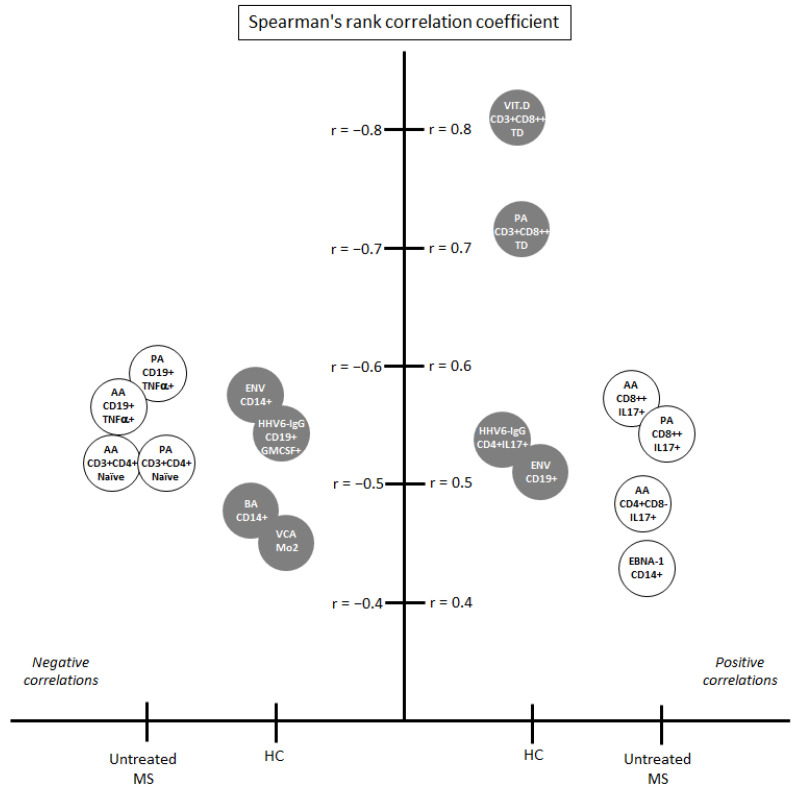
Main correlations among environmental factors and cell subsets: only those with *p* < 0.01 (Mann–Whitney U-test) and Spearman’s rank correlation coefficient (r) above 0.4 or below −0.4 are shown. Gray circles: correlations in HC group; white circles: correlations in MS group.

As we can see in Figure 3, when we analyzed the correlations among the environmental factors included in the study and the panel of immune cells analyzed, we found several positive and negative correlations: 25(OH)D with CD3+CD8++ TD cells (*r* = 0.813; <0.0001), PA with the same cell subset (*r* = 0.721; *p* = 0.0002) and HHV-6 IgG with CD4+CD8-IL17+ cells (*r* = 0.535; *p* = 0.006) were the main positive correlations, and HHV-6 IgG with CD19+GM-CSF+ (*p* = −0.543; *p* = 0.005) was the main negative correlation. All of them had a Spearman’s rank correlation coefficient above 0.5 or below −0.5, respectively.

#### 3.4.2. Correlations with Immune Cells in Untreated MS Patients

As we have previously mentioned, the flow cytometry analysis in MS patients was only performed in those without treatment at sample collection (*n* = 57). As we can see in Figure 3, the main correlations were obtained only with SCFA: AA with CD8++IL17+ cells (*r* = 0.584; *p* = 0.0007) and PA with the same cell subset (*r* = 0.549; *p* = 0.003) were the main positive correlations, and PA with CD19+TNFalpha+ cells (*r* = −0.592; *p* = 0.001), AA with the same cell subset (*r* = −0.558; *p* = 0.002), PA with CD3+CD4+ Naïve cells (*r* = −0.520; *p* = 0.004) and AA with the same cell subset (*r* = −0.520; *p* = 0.003) were the main negative correlations. All of them with a Spearman’s rank correlation coefficient above 0.5 or below −0.5, respectively. None of the correlations found for one of the study groups were found in the others.

**Figure 4:**
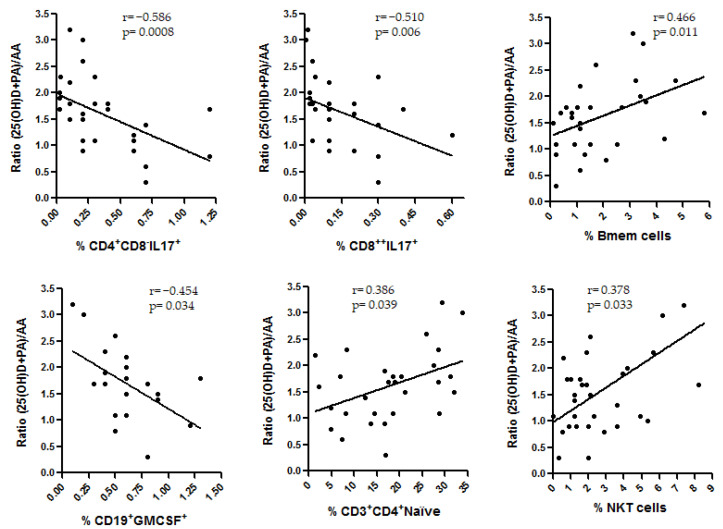
Significant correlations among (25(OH)D+PA)/AA algorithm and the cell subsets analyzed in untreated MS patients; *p*-values (Mann–Whitney U-test) and Spearman’s rank correlation coefficient (r) are shown.

As we can see, 25(OH)D, PA and AA were the environmental factors more frequently involved with the different variables analyzed in this study. In summary: (1) we found correlations for 25(OH)D and AA with the EDSS (opposite effects), (2) a positive correlation between PA/AA ratio and 25(OH)D (Table 4) was also found in MS patients, (3) a positive correlation was found for 25(OH)D and PA with CD8+ T (TD) cells among HC and (4) correlations were also found between PA and AA with IL-17-producing CD8+ T cells (positive correlation) and with TNF-α-producing CD19+ cells and naïve CD4+ T-cells (negative correlation y both cases) in MS patients. Thus, we decided to analyze how the interaction of these variables could be associated with the immune cells in these patients. With this purpose, taking into account the results of this study and those previously published, we built an algorithm similar to the PA/AA ratio with 25(OH)D and PA adding their anti-inflammatory effects: (25(OH)D+PA)/AA (each individual value of each variable was normalized by dividing it by its corresponding median). Then, we analyzed the possible correlations of this algorithm with the cell subsets included in this study as we previously performed for each environmental factor. Figure 4 shows the significant correlations that were found in untreated MS patients. As we can see, the more significant correlations were found between this ratio and the IL-17-producing CD4+ and CD8+ T cells (*r* = −0.586, *p* = 0.0008 and *r* = −0.510, *p* = 0.006, respectively).

## 4. Discussion

Here, we have analyzed several environmental factors that have been previously related to MS pathogenesis. Confirming previous results, we found statistically significant differences for most of them when we compared MS patients and HC. Thus, untreated MS patients showed significantly higher levels of HHV-6A/B IgG (*p* = 0.0005), EBNA-1 IgG (*p* = 0.0004), VCA IgG (*p* = 0.010) and AA (*p* = 0.011) in comparison with HC, but significantly lower levels of 25(OH) (*p* = 0.007) and a significant decrease of the PA/AA and BA/AA ratios (*p* < 0.0001 in both cases). These results would support the possible involvement of these viruses, the vitamin D and the SCFAs in MS etiopathogenesis, as it has been widely published in recent years [1,2,3,4,5,6,7,8,9,10,11,12,13,14,15]. When we analyzed their possible correlations, we found differences between HC and MS patients. The most significant correlations in the MS group were found between HHV-6A/B IgG and IgM titers, among the three SCFA and between the levels of 25(OH)D and the PA/AA ratio—the last is the only correlation found between two environmental factors not clearly related to each other. Then, we analyzed the correlations of these environmental factors with different clinical variables. The strongest correlations were found for the EDSS score and the MSSS: a negative correlation between 25(OH)D with EDSS and MSSS (especially in untreated MS patients), a positive correlation between AA and EDSS (in untreated MS patients) and a negative correlation between HHV-6A/B IgG titers and MSSS (only in treated MS patients). Positive correlations between SCFA and disease duration and negative correlations between these metabolites and the starting age were also found among untreated MS patients.

The negative association of vitamin D with the EDSS has been previously published. Recently, a study that investigated the associations between hypovitaminosis D and disease activity in a cohort of MS patients recruited to the EVIDIMS study (Efficacy of Vitamin D Supplementation in Multiple Sclerosis; NCT01440062) found that EDSS score was negatively associated with the baseline serum vitamin D levels (*p* < 0.001) [21]. This study supports previous publications showing the same correlation between the levels of 25(OH)D and the EDSS score in non-supplemented MS patients in different countries [22,23,24,25,26]. However, a recent meta-analysis that assessed the effects of vitamin D supplementation on the EDSS scores in people with MS in randomized controlled trials found that supplementation with vitamin D alone and vitamin D plus calcium did not affect the EDSS score [27], confirming previous results [28]. Therefore, supplementation with vitamin D would not be able to modify the progression of the disease, despite the correlation described between 25(OH)D levels and EDSS score in non-supplemented MS patients. Some authors try to explain this possible contradiction in some limitations of the meta-analysis that should be taken into consideration, mainly, the heterogeneity of the studies (different dose of vitamin D, study duration, lifestyle habits, etc.) [29], and hypothesized that therapeutic effects in clinically isolated syndrome or MS patients may require longer periods of vitamin D administration [30].

Regarding SCFA, they have been recognized as mediators of immune responses and they could reflect the variations observed in the bacterial composition of the gut microbiota [31]. Recently, a study described that serum and feces of subjects with MS exhibited significantly reduced PA amounts compared with HC, particularly after the first relapse [15]; similarly, another study that analyzed the concentration of BA by LC-MS found that BA was significantly reduced compared to HC [14]. These results do not agree with a previous study of our group [32], nor with the results obtained in this study. As we can see in Figure 2, we did not find any statistically significant difference between the levels of PA and BA in HC and MS patients. However, as we can see in Figure 2A, the ratios PA/AA and BA/AA were significantly higher in HC than in MS patients, and in treated MS patients than in untreated ones. Furthermore, the *p*-values for these ratios were more significant than the *p*-values of each one of the SCFAs, showing a possible opposite role for PA or BA and AA. This could support prior studies that show an anti-inflammatory effect for PA and BA [15,19], while AA would be an activator of the immune cells [20]. These ratios, mainly the PA/AA ratio, could be useful to identify MS patients. In our study, only 4/48 HC had a PA/AA ratio under the median value of the untreated MS patients (0.120) and only 3/49 untreated MS patients had a PA/AA ratio above the median value of the HC (0.208). This should be further studied in larger cohorts. However, although a significant correlation was found between AA levels and EDSS score, we did not find any correlation between PA/AA or BA/AA ratios and the EDSS score or the MSSS. Published supplementation studies performed with PA are short-term studies to evaluate the impact of this therapy on EDSS score [15].

An interesting result of this study is the correlation that we found between 25(OH)D levels and PA/AA ratio (*r* = 0.365; *p* = 0.0003) in MS patients. But what could be the link between vitamin D and the SCFA? One hypothesis could be associated with the role of vitamin D in the regulation of intestinal calcium absorption. It has been described that the hormonally active form of vitamin D, 1,25-dihydroxyvitamin D3 (1,25(OH)2D3), mediates active transport of calcium by distal as well as proximal segments of the intestine [33]. Calcium homeostasis is needed for the contraction-relaxation regulation of the intestinal smooth muscle cells. Previous publications showed that vitamin D seems to increase the synthesis of the calcium channel, suggesting an indirect role in the mechanism of contraction and therefore, stimulating normal intestinal motility [34]. Bowel symptoms (constipation and/or fecal incontinence) affect up to two-thirds of patients with MS [35], and the effects on the end-organ include, among others, alterations of gut motility [36]. MS patients are deficient in vitamin D, and vitamin D deficiency reduces intestinal calcium absorption, leading to gut stasis and subsequently increasing gut permeability [37]. Therefore, gut microbiota can transfer more metabolites, such as the SCFA, into the blood. Since gut microbiota in MS patients is altered [12], different metabolites or different proportions of these metabolites could be found in MS patients and controls, as we have found in our study (Figure 2).

Since environmental factors, like SCFA, could modulate the immune response, we decided to analyze the correlations between them and different cell subsets. These correlations were only performed in HC and in untreated MS patients. The ability of the disease-modifying treatments to change the proportions of the different cells’ subsets has been widely published [38,39,40,41,42,43]. As we can see in Figure 3, we found different statistically significant correlations in both groups. Among the untreated MS patients, only two SCFA were involved in the main correlations: PA and AA, with TNF-α-producing B cells (*r* = −0.592 and *r* = −0.558, respectively) and with IL-17-producing CD8+ T cells (*r* = 0.549 and *r* = 0.584, respectively), showing the importance of the metabolites from gut microbiota in the disease. However, in the HC group, the main correlations were found between the 25(OH)D levels and PA with CD8+ T (TD) cells (*r* = 0.813 and *r* = 0.721, respectively). These results would support the potential beneficial role of both vitamin D and PA in MS. Interestingly, MS patients with acute relapse seem to display a significant loss in CD8+ T (TD) cells, with a concurrent loss in expression of perforin and granzyme B [44]. Furthermore, lower pre-treatment frequencies of CD8+ T (TD) cells were also seen in different cohorts of MS patients [45,46].

As we have previously mentioned, we analyzed how the combination of 25(OH)D, PA and AA could be associated with the immune cells in MS patients. As we can see in Figure 4, those untreated patients with higher levels of 25(OH)D and PA levels in relation to AA levels showed lower frequencies of IL-17-producing CD4+ and CD8+ T cells (*r* = −0.586, *p* = 0.0008 and *r* = −0.510, *p* = 0.006, respectively). Although MS pathogenesis still remains elusive, the TH17 lineage has been repeatedly implicated in the disease [47]. Elevated proportions of TH17 cells and IL-17 have been found in the peripheral blood [48] and CSF [49] of MS patients in comparison with HC. Furthermore, an increased percentage of TH17 cells and mRNA levels of IL-17 were described in brain lesions of MS patients as compared to HC [50]; in fact, an enrichment of IL-17-producing T cells of >70% was found in active MS lesions in comparison to 17% in inactive plaques [51]. Therefore, there are evidences supporting the importance of TH17 cells in MS. Regarding vitamin D, the ability of in vitro 1,25(OH)2D in modulating different Th17 cell subsets in MS patients in remission by attenuating the percentage of pathogenic T cells has been published [52]. Moreover, PA seems to increase the amount of intestine-derived Treg cells [53]. Since TH17 cells and Treg cells share a common precursor cell (the naïve CD4 T cell), the increase of PA levels could lead to a significant reduction in the frequency of TH17 cells [54]. In a study with therapy-naive MS patients, Th17 cells decreased significantly after PA supplementation [14].

In our study, we have described the in vivo interaction between these two environmental factors, the vitamin D and the SCFA, in untreated MS patients. This interaction could have an impact on the pathogenesis of the disease through the TH17 pathway. Furthermore, a recent study shows that rats treated with PA promoted vitamin D receptors (VDR) expression in the intestine via the activity of yes-associated protein (YAP), both in vitro and in vivo [55]. The higher expression of VDRs could increase the effect of vitamin D in the regulation of intestinal calcium absorption and, finally, on gut motility and permeability. This synergistic effect described between vitamin D and PA could be related to the results described in our study (Figure 4). This could have important consequences in terms of their use as possible treatments of the disease. As we have previously mentioned, there are not conclusive results about the improvement of MS through vitamin D supplementation [26,27]. Perhaps, a valuable option to be evaluated in future studies would be the simultaneous modification of these two environmental factors, through the combination of vitamin D supplementation with PA or probiotics administration (to modify the proportion of the SCFA produced by the gut microbiota, increasing the levels of PA or BA and reducing the production of AA). In our study, positive correlations between SCFA and disease duration and negative correlations between the three SCFA and the starting age were also found among untreated MS patients (Supplementary Table S5). However, we did not find any correlation with the age of the patients. These results suggest that there are different mechanisms (probably immunological) or factors (such as hypovitaminosis D among others) associated with the disease that contribute to feedback the dysbiosis in MS patients. A long established dysbiosis would lead to an increasingly different profile of the metabolites from the gut microbiota. Therefore, the early modification of the gut microbiota, such as SCFA levels, could be crucial to modify the progress of the disease.

An intriguing result found in this study is the negative correlation between HHV-6A/B IgG titers and MSSS, only in treated MS patients. Thus, those MS patients with lower HHV-6A/B IgG titers would have a more severe disease than those with higher titers, although different studies support the possible role of this virus in the pathogenesis of the disease [56,57]. Furthermore, in this study, we have described that HHV-6A/B IgG titers were significantly increased when we compared untreated MS patients and HC (*p* = 0.0005; Figure 2). However, we did not find any statistically significant difference between treated MS patients and HC (Figure 2). What could be the explanation? HHV-6A/B has been associated with the relapses [3], and with the inflammatory status of the disease [58]. More active MS patients are those with higher relapse rates and usually, they have lower EDSS scores; therefore, HHV-6A/B IgG titers would be higher in these patients and a negative correlation between them and the progression or the severity of the disease could be found. In this study, as we can see, in Supplementary Figure S1, that HHV-6A/B IgG titers were not statistically significantly different between SPMS patients and HC, while the *p*-value was even more significant when we compared RRMS patients and HC (*p* = 0.0008) than when we compared the whole population of MS patients with the HC (*p* = 0.003). This is a cross-sectional study and we have not analyzed the relation between the evolution of the HHV-6A/B IgG titers and the progression of the disease in each patient, as we have done in other longitudinal studies [3,59]. Finally, since a paper analyzing HHV-6A and HHV-6B in MS patients has been recently published using a novel multiplex serological assay, it would be very interesting for future studies to know if the observed negative correlation between IgG titers and MSSS in treated MS patients is due to one of these two viruses or not [56]. Furthermore, in the same study, the authors found that IgG responses against HHV-6A/B proteins were associated with different HLA haplotypes: while the strongest association for the immediate-early protein 1 from HHV-6A (IE1A) was the DRB1*13:01-DQA1*01:03-DQB1*06:03 haplotype, the main association for the immediate-early protein 1 from HHV-6B (IE1B) was DRB1*13:02-DQA1*01:02-DQB1*06:04. However, the associations described here did not differ between MS subtypes or vary with severity of disease.

When we analyzed the possible correlation between HHV-6A/B IgG titers and the cell subsets, we did not find any significant result among the untreated MS patients, but we did in the HC group: HHV-6A/B IgG titers correlated with IL-17-producing CD4+ T cells (*r* = 0.535) and with GM-CSF-producing B cells (*r* = −0.543) (Figure 3). These correlations would support its possible contribution to the pathology of MS. We have previously mentioned the involvement of TH17 lineage in the disease. Furthermore, the membrane co-factor protein CD46, the cellular receptor for a number of pathogens including the HHV-6A/B, would influence T-cell activation [58]. The CD3/CD46 cross-linking would induce expression of IL-1beta and IL-17A in MS patients’ T cells, suggesting a potential mechanism of virus-induced neuroinflammation that could be involved in MS disease pathogenesis. The role of GM-CSF in MS is still not fully elucidated. It is known that its concentration is increased in the cerebrospinal fluid of patients with active MS compared to HC, but more mechanistic studies are needed to address the role of GM-CSF-producing B and T cells in MS [60]. However, the role of GM-CSF has been studied in animal models. One study suggests that GM-CSF signaling is compulsory for the progression in the experimental autoimmune encephalomyelitis (EAE) model [61]. Further studies will be needed to study if the negative association described here between HHV-6A/B IgG titers and GM-CSF-producing B cells and MSSS could be related to the possible involvement of GM-CSF in the progression of MS.

Statistically significant correlations were also found between CMV IgG titers and the age of the MS patients (positive correlation; Supplementary Table S2). It is known that CMV seroprevalence increases with age [62]. Furthermore, CMV has been shown to accelerate immune ageing by affecting peripheral blood T cell phenotypes and increasing inflammatory mediated cytokines [63]. Premature aging could be a risk factor for developing autoimmune disorders like MS [64]. However, different serological studies suggest that CMV is associated with a decrease in the risk of MS. In a multiethnic study performed in Southern California, authors found that CMV seropositivity was associated with a lower risk of MS/CIS in Hispanics (*p* = 0.004) but not in blacks or whites [65]. In another study, CMV infection was found more common in children with monophasic acquired demyelinating syndromes than with MS [66]. A previous study in the pediatric population had shown that a remote infection with CMV was associated with a lower risk of developing MS (*p* = 0.004) after multivariate analysis [67]. In our study, MS patients had lower prevalence of CMV IgG antibodies (Table 1; *p* = 0.011) and lower CMV IgG titers (Figure 2A; *p* = 0.004) than HC. Therefore, further studies are needed to understand the intriguing relationship between CMV and MS.

Finally, a statistically significant correlation was also found between VCA IgG titers and the starting age, only among treated MS patients (negative correlation; Supplementary Table S4). Although EBV has been repeatedly associated with MS [2,9,65], and we have found statistically significant differences in the prevalence of EBNA-1 IgG between MS patients an HC, and in the EBNA-1 and VCA IgG titers between untreated MS patients and HC, we have not found any correlation between them and the other environmental factors, with the clinical variables analyzed or with the immune cells in our study. The only correlation found is the one mentioned above: higher VCA IgG titers were found in those MS patients with a lower age of onset. EBV infection has been associated with early MS, and different studies support the concept that EBV could be a trigger for MS, acting very early in the development of the disease [68].

One limitation of the study is the absence of information related to the smoking status of both MS patients and HC. As we have previously mentioned in the introduction, smoking is other environmental factor related to MS pathogenesis in the last years. Cigarette smoking can induce epigenetic modifications in blood cells that can change the expression levels of different genes, and these changes seem to be larger in patients with the major genetic risk factors for MS [69]. Furthermore, a recent review showed that part of the severity, progression and early death in MS could be explained in significant part by the immunological, proinflammatory effects of smoking [70]. Therefore, further studies analyzing interactions between environmental factors and immune cells should include smoking status.

## 5. Conclusions

In summary, we found statistically significant differences for most of the environmental factors analyzed when we compared MS patients and HC, supporting their possible involvement in the disease. However, the strongest correlations with the clinical variables and the cell subsets analyzed were found for 25(OH)D and SCFA levels. Furthermore, a correlation was found between these two environmental factors, and their interaction negatively correlated with IL-17-producing CD4+ and CD8+ T cells in untreated MS patients. Since TH17 cells have been implicated in the pathogenesis of MS, intervention studies increasing vitamin D levels and modifying the proportion of SCFA at the same time could be evaluated in the future.

## Figures and Tables

**Table 1:** Demographical characteristics of the relapsing-remitting multiple sclerosis (RRMS) patients and healthy controls included in the study at the sample collection.

	MS Patients	Controls
Females (*n* (%))	129 (67.5%)	50 (63.3%)
Age (years, med (P25–P75))	41.0 (35.0–47.5)	40.0 (34.0–45.5)
Age at disease onset (years, med (P25–P75))	30.0 (25.0–36.0)	N/A
Disease duration at recruitment (months, med (P25–P75))	106.0 (56.5–177.5)	N/A
MS type: RRMS (*n* (%))	133 (69.6%)	N/A
EDSS (med (P25–P75))	2.0 (1.0–5.5)	N/A
MSSS (med (P25–P75))	2.6 (0.9–6.0)	N/A
ARR since the beginning of the disease (med (P25–P75)))	0.6 (0.3–1.1)	N/A
Number of relapses two years before (med (P25–P75)))	1.0 (0.0–2.0)	N/A
Non-treated MS patients at recruitment (*n* (%))	57 (29.8%)	N/A
Treatment-naïve (n)	21	N/A
Last treatment for non-naïve (months, med (P25–P75))	2.5 (1.0–3.0)	N/A
Treated MS patients at recruitment (*n* (%))	134 (70.2%)	N/A
MS patients treated with interferon beta	51	N/A
MS patients treated with glatiramer acetate	33	N/A
MS patients treated with natalizumab	33	N/A
MS patients treated with fingolimod	8	N/A
MS patients treated with teriflunomide	5	N/A
MS patients treated with dimethyl fumarate	4	N/A
Treatment duration (med (P25–P75))	25.0 (11.0–53.0)	N/A
Patients with 9 or more T2 lesions at recruitment (*n* (%)) *	135 (91.2%)	N/A
Patients with Gd+ lesions at recruitment (*n* (%)) **	26 (20.5%)	N/A

med: median; P25: 25th percentile; P75: 75th percentile; N/A: not applicable; EDSS: Expanded Disability Status Scale; MSSS: Multiple Sclerosis Severity Score; ARR: annualized relapse rate; Gd+: Gadolinium enhancing lesions. * Only for 148 MS patients do we have data of T2 lesions (±3 months around the sample collection). ** Only for 127 MS patients do we have data of gadolinium enhancing lesions (±3 months around the sample collection).

**Table 2:** IgG and IgM prevalence of the viruses included in the study.

	HHV-6A/B IgG	HHV-6A/B IgM	EBNA-1 IgG	VCA IgG	CMV IgG	CMV IgM
MS	160/182 (87.9%)	25/179 (14.0%)	176/186 (94.6%)	184/185 (99.5%)	105/171 (61.4%)	8/135 (5.9%)
HC	52/60 (86.7%)	5/62 (8.1%)	54/63 (85.7%)	60/63 (95.2%)	37/45 (82.2%)	0/35 (0%)
*p*	0.800	0.231	**0.027**	0.057	**0.011**	0.289
OR * (95% CI) **	1.12 (0.47–2.66)	1.85 (0.68–5.07)	2.93 (1.13–7.59)	9.2 (0.94–90.12)	2.91 (1.28–6.63)	4.73 (0.27–84.02)

Those doubtful values, between 9 and 11 AU, where excluded. * *p*-values were calculated from Chi-square test/Fisher’s exact test. ** Odds Ratios (OR) with the 95% Confidence Intervals (CI). Bold values indicate the statistically significant values.

**Table 3:** Correlations among the environmental factors analyzed in samples collected from healthy controls.

	HHV-6 A/B IgG	HHV-6 A/B IgM	EBNA-1 IgG	VCA IgG	CMV IgG	CMV IgM	25(OH)D	AA	PA	BA	PA/AA	BA/AA
HHV6A/B IgG ^1^		*r* = 0.176 n.s.	*r* = 0.123 n.s.	*r* = 0.180 n.s.	*r* = 0.145 n.s.	*r* = 0.087 n.s.	*r* = −0.008 n.s.	*r* = −0.058 n.s.	*r* = −0.024 n.s.	*r* = −0.202 n.s.	*r* = −0.017 n.s.	*r* = −0.119 n.s.
HHV6A/B IgM ^1^			*r* = −0.053 n.s.	*r* = −0.030 n.s.	*r* = 0.371 *p* = 0.026	***r*** = **0.623** ***p* < 0.0001**	*r* = 0.265 *p* = 0.048	*r* = 0.028 n.s.	*r* = −0.046 n.s.	*r* = −0.080 n.s.	*r* = −0.028 n.s.	*r* = −0.148 n.s.
EBNA1 IgG ^1^				*r* = 0.114 n.s.	*r* = 0.214 n.s.	*r* = −0.065 n.s.	*r* = −0.087 n.s.	*r* = −0.175 n.s.	*r* = −0.295 *p* = 0.044	*r* = −0.183 n.s.	*r* = −0.145 n.s.	*r* = −0.116 n.s.
VCA IgG ^1^					*r* = 0.138 n.s.	*r* = −0.066 n.s.	*r* = 0.007 n.s.	*r* = −0.214 n.s.	*r* = −0.131 n.s.	*r* = −0.124 n.s.	*r* = −0.096 n.s.	*r* = 0.176 n.s.
CMV IgG ^1^						*r* = 0.320 n.s.	*r* = 0.170 n.s.	*r* = −0.026 n.s.	*r* = −0.107 n.s.	*r* = 0.020 n.s.	*r* = −0.023 n.s.	*r* = 0.071 n.s.
CMV IgM ^1^							*r* = 0.003 n.s.	*r* = 0.175 n.s.	*r* = −0.161 n.s.	*r* = −0.028 n.s.	*r* = −0.277 n.s.	*r* = −0.288 n.s.
25(OH)D ^2^								*r* = −0.142 n.s.	*r* = 0.242 n.s.	*r* = 0.203 n.s.	*r* = 0.370 *p* = 0.011	*r* = 0.406 *p* = 0.009
AA ^3^									***r*** = **0.450** ***p* = 0.001**	*r* = 0.364 *p* = 0.016	*r* = 0.364 *p* = 0.016	***r*** = **−0.482** ***p* = 0.0008**
PA ^3^										***r*** = **0.468** ***p* = 0.003**	***r*** = **0.468** ***p* = 0.003**	*r* = 0.114 n.s.
BA ^3^											*r* = −0.016 n.s.	***r*** = **0.570** ***p* < 0.0001**
PA/AA												***r*** = **−0.549** ***p* = 0.0002**
BA/AA												

Correlations were assessed by using the Spearman’s rank correlation coefficient (r). Bold values indicate the statistically significant values after Bonferroni correction (*p* < 0.004); significant *p*-values prior to Bonferroni correction are also shown. Results were obtained as: ^1^ artificial units (AU), ^2^ ng/Ml and ^3^ μmol/L. n.s.: not significant.

**Table 4:** Correlations among the environmental factors analyzed in samples collected from MS patients.

	HHV-6 A/B IgG	HHV-6 A/B IgM	EBNA-1 IgG	VCA IgG	CMV IgG	CMV IgM	25(OH)D	AA	PA	BA	PA/AA	BA/AA
HHV6A/BIgG ^1^		***r*** = **0.272** ***p* = 0.0002**	*r* = 0.151 *p* = 0.039	*r* = 0.124 n.s.	*r* = 0.013 n.s.	*r* = 0.127 n.s.	*r* = 0.226 *p* = 0.023	*r* = −0.155 n.s.	*r* = 0.007 n.s.	*r* = 0.030 n.s.	*r* = 0.056 n.s.	*r* = 0.093 n.s.
HHV6A/BIgM ^1^			*r* = 0.004 n.s.	*r* = 0.088 n.s.	*r* = 0.011 n.s.	*r* = 0.103 n.s.	*r* = 0.219 *p* = 0.028	*r* = −0.093 n.s.	*r* = 0.152 n.s.	*r* = 0.017 n.s.	*r* = 0.181 n.s.	*r* = 0.146 n.s.
EBNA1IgG ^1^				*r* = 0.153 *p* = 0.036	*r* = 0.069 n.s.	*r* = −0.008 n.s.	*r* = 0.039 n.s.	*r* = −0.014 n.s.	*r* = −0.103 n.s.	*r* = −0.034 n.s.	*r* = −0.107 n.s.	*r* = −0.046 n.s.
VCAIgG ^1^					*r* = 0.062 n.s.	*r* = 0.082 n.s.	*r* = 0.091 n.s.	*r* = 0.004 n.s.	*r* = 0.154 n.s.	*r* = 0.092 n.s.	*r* = 0.201 n.s.	*r* = 0.268 *p* = 0.012
CMVIgG ^1^						*r* = 0.076 n.s.	*r* = −0.086 n.s.	*r* = −0.085 n.s.	*r* = 0.138 n.s.	*r* = −0.014 n.s.	*r* = 0.172 n.s.	*r* = 0.068 n.s.
CMVIgM ^1^							*r* = 0.116 n.s.	*r* = 0.129 n.s.	*r* = 0.105 n.s.	*r* = 0.090 n.s.	*r* = 0.107 n.s.	*r* = 0.107 n.s.
25(OH)D ^2^								*r* = −0.216 *p* = 0.035	*r* = 0.167 n.s.	*r* = 0.129 n.s.	***r*** = **0.365** ***p* = 0.0003**	*r* = −0.235 *p* = 0.029
AA ^3^									***r*** = **0.456** ***p* < 0.0001**	***r*** = **0.395** ***p* = 0.0002**	***r*** = **−0.386** ***p* = 0.0001**	***r*** = **−0.333** ***p* = 0.002**
PA ^3^										***r*** = **0.697** ***p* < 0.0001**	***r*** = **0.527** ***p* < 0.0001**	*r* = 0.247 *p* = 0.023
BA ^3^											*r* = 0.260 *p* = 0.017	***r*** = **0.530** ***p* < 0.0001**
PA/AA												***r*** = **0.505** ***p* < 0.0001**
BA/AA												

Correlations were assessed by using the Spearman’s rank correlation coefficient (r). Bold values indicate the statistically significant values after Bonferroni correction (*p* < 0.004); significant *p*-values prior to Bonferroni correction are also shown. Results were obtained as: ^1^ artificial units (AU), ^2^ ng/Ml and ^3^ μmol/L. n.s.: not significant.

## Data Availability

The data presented in this study are available on request from the corresponding author. The data are not publicly available due to ethical reasons.

## Supplementary Materials

The following are available online at https://www.mdpi.com/2073-4409/10/1/119/s1, Table S1: Study of the possible association of the environmental factors analyzed with the gender and age of the HC; Table S2: Study of the possible association of the environmental factors analyzed with the gender and age of the MS patients; Table S3: Correlations between the environmental factors included in the study and clinical variables in MS patients group; Table S4: Correlations between the environmental factors included in the study and clinical variables in treated MS patients; Table S5: Correlations between the environmental factors included in the study and clinical variables in untreated MS patients; Figure S1: Comparisons of the environmental factors analyzed between HC and RRMS and SPMS patients.
