# Supplementary material for: Herpesvirus Antibodies, Vitamin D and Short-Chain Fatty Acids: Their Correlation with Cell Subsets in Multiple Sclerosis Patients and Healthy Controls
Source: Cells. 2021 Jan 10;10(1):119. doi: 10.3390/cells10010119 (PMC7826528; doi:10.3390/cells10010119)
Supplement: Supplementary file 1 [file cells-10-00119-s001.zip › Supplementary Material_Table S2.pdf]

Table S2. Study of the possible association of the environmental factors analyzed with the gender and age of the MS patients.

	GENDER			AGE
	**Male (median)**	**Female (median)**	**p**	
**HHV6-A/B IgG[1]**	26.1	34.1	0.065	r=-0.084 n.s.
**HHV6-A/B IgM[1]**	3.2	4.9	**0.033**	r=-0.059 n.s.
**EBNA-1IgG[1]**	26.0	26.0	0.170	r=0.113 n.s.
**VCA IgG[1]**	51.0	54.0	0.333	r=-0.102 n.s.
**CMV IgG[1]**	18.2	21.5	0.374	**r=0.255 p=0.002**
**CMV IgM[1]**	3.6	3.0	0.804	r=-0.025 n.s.
**25(OH)D[2]**	18.8	20.0	0.581	r=-0.090 n.s.
**AA[3]**	23.5	27.9	0.319	r=0.082 n.s.
**PA[3]**	3.8	3.6	0.840	r=0.105 n.s.
**BA[3]**	3.7	4.2	0.517	r=0.020 n.s.
**PA/AA**	0.134	0.139	0.297	r=0.068 n.s.
**BA/AA**	0.148	0.126	0.433	r=-0.108 n.s.

Significations with the two-tailed t-test for the possible association of the environmental factors with the gender are shown. Correlations between environmental factors and age were assessed by using the Spearman's rank correlation coefficient (r). Bold values indicates the statistically significant values. Results were obtained as: [1] artificial units (AU), [2] ng/Ml and [3] µmol/L. (n.s.: not significant).
