# Supplementary material for: Herpesvirus Antibodies, Vitamin D and Short-Chain Fatty Acids: Their Correlation with Cell Subsets in Multiple Sclerosis Patients and Healthy Controls
Source: Cells. 2021 Jan 10;10(1):119. doi: 10.3390/cells10010119 (PMC7826528; doi:10.3390/cells10010119)
Supplement: Supplementary file 1 [file cells-10-00119-s001.zip › Supplementary Material_Table S3.pdf]

Table S3. Correlations between the environmental factors included in the study and clinical variables in MS patients group.

	Starting age (years)	Disease duration (months)	EDSS	MSSS	ARR	Relapses 2-years earlier
**HHV-6A/B IgG[1]**	r=-0.120 n.s.	r=0.025 n.s.	r=-0.170 p=0.021	**r=-0.198 p=0.007**	r=-0.079 n.s.	r=-0.128 n.s.
**HHV-6A/B IgM[1]**	r=0.001 n.s.	r=-0.113 n.s.	r=-0.181 p=0.014	r=-0.147 p=0.047	r=-0.157 n.s.	r=-0.025 n.s.
**EBNA-1 IgG[1]**	r=0.111 n.s.	r=-0.046 n.s.	r=-0.012 n.s.	r=0.017 n.s.	r=0.067 n.s.	r=0.110 n.s.
**VCA IgG[1]**	r=0.163 p=0.025	r=-0.026 n.s.	r=-0.082 n.s.	r=-0.060 n.s.	r=-0.101 n.s.	r=-0.126 n.s.
**CMV IgG[1]**	r=0.174 p=0.040	r=0.093 n.s.	r=0.131 n.s.	r=0.132 n.s.	r=0.113 n.s.	r=-0.105 n.s.
**CMV IgM[1]**	r=0.015 n.s.	r=-0.100 n.s.	r=-0.151 n.s.	r=-0.145 n.s.	r=-0.145 n.s.	r=-0.138 n.s.
**25(OH)D[2]**	r=-0.055 n.s.	r=-0.020 n.s.	**r=-0.372 p=0.0001**	**r=-0.402 p<0.0001**	r=0.150 n.s.	r=0.115 n.s.
**AA[3]**	r=-0.181 n.s.	**r=0.309 p=0.002**	**r=0.363 p=0.0003**	r=0.240 p=0.018	r=-0.231 n.s.	r=-0.194 n.s.
**PA[3]**	r=-0.162 n.s.	r=0.253 p=0.014	r=0.067 n.s.	r=-0.020 n.s.	r=-0.139 n.s.	r=-0.112 n.s.
**BA[3]**	r=-0.145 n.s.	r=0.217 p=0.048	r=0.081 n.s.	r=0.016 n.s.	r=-0.104 n.s.	r=-0.058 n.s.
**PA/AA**	r=-0.030 n.s.	r=0.034 n.s.	r=-0.202 p=0.048	r=-0.220 p=0.031	r=-0.017 n.s.	r=0.006 n.s.
**BA/AA**	r=-0.028 n.s.	r=-0.106 n.s.	r=-0.262 p=0.014	r=-0.241 p=0.024	r=-0.021 n.s.	r=-0.019 n.s.

Correlations were assessed by using the Spearman's rank correlation coefficient (r). Bold values indicates the statistically significant values after Bonferroni correction (p<0.008); significant p values prior Bonferroni correction are also shown. Results were obtained as: [1] artificial units (AU), [2] ng/mL and [3] μmol/L. (n.s.: not significant).
