# Supplementary material for: Herpesvirus Antibodies, Vitamin D and Short-Chain Fatty Acids: Their Correlation with Cell Subsets in Multiple Sclerosis Patients and Healthy Controls
Source: Cells. 2021 Jan 10;10(1):119. doi: 10.3390/cells10010119 (PMC7826528; doi:10.3390/cells10010119)
Supplement: Supplementary file 1 [file cells-10-00119-s001.zip › Supplementary Material_Table S4.pdf]

Table S4. Correlations between the environmental factors included in the study and clinical variables in treated MS patients.

	**Starting age (years)**	**Disease duration (months)**	**EDSS**	**MSSS**	**ARR**	**Relapses 2-years earlier**
**HHV-6A/B IgG[1]**	r=-0.207 p=0.018	r=0.046 n.s.	r=-0.218 p=0.014	**r=-0.264 p=0.003**	r=-0.130 n.s.	r=-0.181 n.s.
**HHV-6A/B IgM[1]**	r=-0.088 n.s.	r=0.054 n.s.	r=-0.166 n.s.	r=-0.157 n.s.	r=-0.186 n.s.	r=-0.059 n.s.
**EBNA-1 IgG[1]**	r=-0.101 n.s.	r=-0.021 n.s.	r=-0.002 n.s.	r=0.007 n.s.	r=0.044 n.s.	r=-0.134 n.s.
**VCA IgG[1]**	**r=-0.238 p=0.006**	r=0.046 n.s.	r=0.014 n.s.	r=0.003 n.s.	r=-0.125 n.s.	r=-0.136 n.s.
**CMV IgG[1]**	r=0.124 n.s.	r=0.113 n.s.	r=0.132 n.s.	r=0.106 n.s.	r=0.076 n.s.	r=0.113 n.s.
**CMV IgM[1]**	r=-0.002 n.s.	r=-0.118 n.s.	r=-0.185 n.s.	r=-0.168 n.s.	r=-0.157 n.s.	r=-0.142 n.s.
**25(OH)D[2]**	r=-0.186 n.s.	r=0.032 n.s.	r=-0.280 p=0.041	r=-0.319 p=0.019	r=-0.024 n.s.	r=0.053 n.s.
**AA[3]**	r=0.146 n.s.	r=0.329 p=0.022	r=-0.292 p=0.044	r=-0.211 n.s.	r=-0.272 n.s.	r=-0.247 n.s.
**PA[3]**	r=0.074 n.s.	r=0.181 n.s.	r=-0.028 n.s.	r=-0.070 n.s.	r=-0.156 n.s.	r=-0.137 n.s.
**BA[3]**	r=0.106 n.s.	r=0.076 n.s.	r=-0.165 n.s.	r=-0.165 n.s.	r=-0.129 n.s.	r=-0.097 n.s.
**PA/AA**	r=0.021 n.s.	r=0.021 n.s.	r=-0.171 n.s.	r=-0.183 n.s.	r=-0.030 n.s.	r=0.003 n.s.
**BA/AA**	r=0.034 n.s.	r=-0.148 n.s.	r=-0.323 p=0.045	r=-0.266 n.s.	r=-0.014 n.s.	r=-0.008 n.s.

Correlations were assessed by using the Spearman's rank correlation coefficient (r). Bold values indicates the statistically significant values after Bonferroni correction ($p<0.008$); significant p values prior Bonferroni correction are also shown. Results were obtained as: [1] artificial units (AU), [2] ng/Ml and [3] µmol/L. (n.s.: not significant).
