# Supplementary material for: Herpesvirus Antibodies, Vitamin D and Short-Chain Fatty Acids: Their Correlation with Cell Subsets in Multiple Sclerosis Patients and Healthy Controls
Source: Cells. 2021 Jan 10;10(1):119. doi: 10.3390/cells10010119 (PMC7826528; doi:10.3390/cells10010119)
Supplement: Supplementary file 1 [file cells-10-00119-s001.zip › Supplementary Material_Table S5.pdf]

Table S5. Correlations between the environmental factors included in the study and clinical variables in untreated MS patients.

	**Starting age (years)**	**Disease duration (months)**	**EDSS**	**MSSS**	**ARR**	**Relapses 2-years earlier**
**HHV-6A/B IgG[1]**	r=-0.122 n.s.	r=-0.052 n.s.	r=-0.164 n.s.	r=-0.135 n.s.	r=0.171 n.s.	r=0.098 n.s.
**HHV-6A/B IgM[1]**	r=0.136 n.s.	r=-0.176 n.s.	r=-0.153 n.s.	r=-0.074 n.s.	r=0.114 n.s.	r=-0.091 n.s.
**EBNA-1 IgG[1]**	r=-0.195 n.s.	r=-0.020 n.s.	r=-0.009 n.s.	r=-0.010 n.s.	r=-0.143 n.s.	r=-0.134 n.s.
**VCA IgG[1]**	r=-0.030 n.s.	r=-0.128 n.s.	r=-0.190 n.s.	r=-0.150 n.s.	r=-0.086 n.s.	r=-0.070 n.s.
**CMV IgG[1]**	r=0.137 n.s.	r=-0.042 n.s.	r=0.128 n.s.	r=0.183 n.s.	r=0.043 n.s.	r=-0.039 n.s.
**CMV IgM[1]**	r=0.126 n.s.	r=-0.027 n.s.	r=0.160 n.s.	r=0.153 n.s.	r=-0.143 n.s.	r=-0.027 n.s.
**25(OH)D[2]**	r=0.082 n.s.	r=-0.078 n.s.	**r=-0.417 p=0.003**	**r=-0.465 p=0.0009**	r=0.113 n.s.	r=0.065 n.s.
**AA[3]**	**r=-0.386 p=0.006**	r=0.364 p=0.010	**r=0.393 p=0.005**	r=0.225 n.s.	r=-0.198 n.s.	r=-0.206 n.s.
**PA[3]**	**r=-0.429 p=0.003**	**r=0.446 p=0.002**	r=0.308 p=0.037	r=0.144 n.s.	r=-0.161 n.s.	r=-0.098 n.s.
**BA[3]**	**r=-0.395 p=0.007**	r=0.357 p=0.016	r=0.260 n.s.	r=0.117 n.s.	r=-0.117 n.s.	r=-0.101 n.s.
**PA/AA**	r=0.001 n.s.	r=0.043 n.s.	r=-0.007 n.s.	r=-0.023 n.s.	r=-0.006 n.s.	r=0.011 n.s.
**BA/AA**	r=-0.002 n.s.	r=-0.099 n.s.	r=-0.090 n.s.	r=-0.100 n.s.	r=-0.038 n.s.	r=-0.014 n.s.

Correlations were assessed by using the Spearman's rank correlation coefficient (r). Bold values indicates the statistically significant values after Bonferroni correction ($p<0.008$); significant p values prior Bonferroni correction are also shown. Results were obtained as: [1] artificial units (AU), [2] ng/Ml and [3] µmol/L. (n.s.: not significant).
