# Supplementary figures and images for: Herpesvirus Antibodies, Vitamin D and Short-Chain Fatty Acids: Their Correlation with Cell Subsets in Multiple Sclerosis Patients and Healthy Controls
Source: Cells. 2021 Jan 10;10(1):119. doi: 10.3390/cells10010119 (PMC7826528; doi:10.3390/cells10010119)

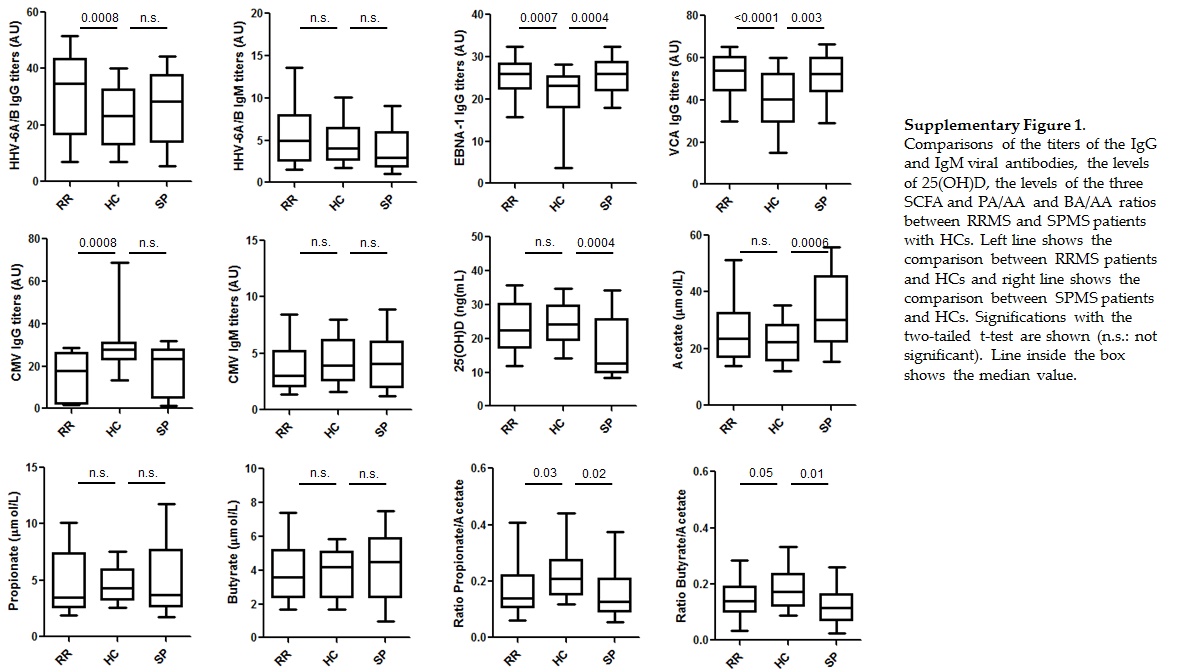

Supplement: Supplementary file 1 [file cells-10-00119-s001.zip › Supplementary Material_Figure S1.jpg]
